# STOP-CINV Study: Safety and Efficacy of IV Akynzeo® (Fosnetupitant 235 mg and Palonosetron 0.25 mg) for the Prevention of Chemotherapy-Induced Nausea and Vomiting in Indian Patients

**DOI:** 10.7759/cureus.91965

**Published:** 2025-09-10

**Authors:** Pragya Shukla, Shaunak Valame, Siddhartha Nanda, Naval K Shakya, Arun K Verma, Abhinandan Hanji, Amullya Pednekar, Sagar Bhagat, Saiprasad Patil, Anup U Petare, Sumit Bhushan, Hanmant Barkate

**Affiliations:** 1 Radiation Oncology, Delhi State Cancer Institute, Delhi, IND; 2 Medical Oncology, Jawaharlal Nehru Cancer Hospital & Research Centre, Bhopal, IND; 3 Radiation Oncology, All India Institute of Medical Sciences, Raipur, Raipur, IND; 4 Medical Oncology, Lakshya Cancer Hospital and Research Centre, Lucknow, IND; 5 Radiation Oncology, Subharti Medical College and Hospital, Meerut, IND; 6 Medical Oncology, Hanji Cancer Hospital, Belagavi, IND; 7 Global Medical Affairs, Glenmark Pharmaceuticals Limited, Mumbai, IND

**Keywords:** antiemetic, cinv, fosnetupitant, nausea, nepa, netupitant, nk1 ra, palonosetron, vomiting

## Abstract

Purpose

IV Akynzeo^®^ (IV fosnetupitant + palonosetron) is the first fixed IV combination designed to target key pathways of emesis, enabling convenient single-dose administration. This study aimed to evaluate the safety and effectiveness of IV Akynzeo^®^ in a real-world context in India.

Methods

This open-label, single-arm, multicenter, prospective phase IV trial assessed a single dose of IV Akynzeo^®^ for the prevention of CINV in patients receiving highly emetogenic or moderately emetogenic chemotherapy (HEC/MEC). IV Akynzeo^®^ (fosnetupitant 235 mg and palonosetron 0.25 mg) was administered over 30 minutes before the start of chemotherapy. The primary endpoints were the number of patients with drug-related treatment-emergent adverse events (TEAEs) as assessed by the treating physician and the number of patients with TEAEs and serious TEAEs over a period of 10 days (±2 days). The key secondary endpoints were complete response, protection, and control in the acute phase (up to 24 hours), delayed phase (>24-120 hours), extended phase (>120-240 hours), overall phase (0-120 hours), and extended overall phase (0-240 hours).

Results

A total of 178 patients were enrolled (median age, 48.5 years; 64% male), of whom 176 completed the study. IV Akynzeo^®^ was well tolerated, with 17 patients (9.55%) reporting 23 adverse events (22 mild and one fatal). No new safety signals were detected. Headache, fatigue, and fever were the most commonly reported AEs. Injection site reactions with IV Akynzeo^®^ were mild in three patients. The complete response rates with IV Akynzeo^®^ were as follows: 84.27% (95% CI: 78.01-89.29) in the acute phase, 93.26% (95% CI: 88.52-96.47) in the delayed phase, 96.07% (171 of 178 patients; 95% CI: 92.07-98.40) in the extended phase, 83.15% (95% CI: 76.82-88.33) in the overall phase, and 80.34% (143 of 178 patients; 95% CI: 73.73-85.91) in the extended overall phase.

Conclusions

IV Akynzeo^®^ was well tolerated and demonstrated substantial effectiveness in mitigating CINV in patients undergoing HEC/MEC across the acute, delayed, and extended phases.

## Introduction

Chemotherapy-induced nausea and vomiting (CINV) is a significant concern for patients, with severe uncontrolled cases being likened to near-death experiences [1-4]. The occurrence of CINV is linked to underlying pathophysiological mechanisms involving both peripheral and central nervous system pathways, mediated by serotonin via 5-hydroxytryptamine receptors and substance P via neurokinin-1 receptors, respectively [4-6]. Acute CINV (within 24 hours after chemotherapy administration) and delayed CINV (>24-120 hours) are characterized by distinct mechanisms: serotonin mediates acute CINV, while substance P is widely recognized as the primary neurotransmitter implicated in delayed CINV [6,7].

CINV can significantly affect daily life in about 50% of patients who experience either acute or delayed CINV and in 90% of patients who experience both acute and delayed CINV [8,9]. Moreover, CINV may persist longer than expected: acute CINV affects up to 55.3% of patients, delayed CINV affects 62.3%, and CINV persisting beyond the expected period affects 36% [10]. The severity of nausea in the extended phase (>120 hours) is comparable to that in the delayed phase. Patients who develop delayed CINV, particularly on Days 4-5, are at increased risk of extended CINV. Although extended-phase CINV is less prevalent than delayed CINV, it still affects a significant number of patients with a similar degree of severity [10].

NEPA (netupitant + palonosetron) is the only fixed antiemetic combination that consists of netupitant, a highly selective and long-lasting NK1 receptor antagonist (RA), and palonosetron, a second-generation 5-HT3RA that is distinct both pharmacologically and clinically. This unique combination provides at least five days of CINV prevention with a single dose, offering an opportunity to enhance adherence to guidelines [11]. The oral formulation of NEPA was approved by the drug regulatory authority of India in 2018 [12]. A fixed IV formulation of NEPA (IV NEPA), using fosnetupitant (a water‐soluble phosphorylated prodrug of netupitant) and palonosetron, was developed to provide additional ease and efficiency. It was approved by the US FDA in 2018 and by the European Medicines Agency in 2020 [13], with a safety and efficacy profile comparable to that of oral NEPA, based on evidence from a multinational, randomized, double-blind, parallel-group, phase III trial [11]. Beyond convenience and efficiency, the IV formulation offers an important benefit for cancer patients who cannot tolerate oral treatments or have swallowing difficulties [13,14]. Current antiemetic guidelines propose that IV NEPA can be used interchangeably with oral NEPA to improve the management of CINV, offering added convenience for patients and practical benefits for clinicians [13].

This study aimed to evaluate the safety and effectiveness of IV Akynzeo^®^ in accordance with the Central Drugs Standard Control Organisation phase IV condition in Form CT-20 for fosnetupitant and palonosetron (235 mg/0.25 mg solution for infusion) in the prevention of CINV in Indian patients receiving highly emetogenic chemotherapy (HEC) or moderately emetogenic chemotherapy (MEC) regimens. To the best of our knowledge, no study with IV Akynzeo^®^ has been conducted in Indian patients. Hence, there is a clinical need to generate real-world evidence on the use of IV Akynzeo^®^ in Indian settings.

## Materials and methods

Study design

This was an open-label, single-arm, multicenter, prospective phase IV study conducted to evaluate the safety and effectiveness of IV Akynzeo^®^ (concentrate for solution for infusion of fosnetupitant 235 mg and palonosetron 0.25 mg) in the prevention of CINV in Indian patients (STOP-CINV study) from May 2023 to October 2023. The study was initiated after approval from the institutional ethics committee (CTRI/2023/04/051951). Demographic data were collected, and informed consent was obtained from each patient prior to enrollment and before any study-specific procedures were performed.

Patients were enrolled across six sites in India after a detailed medical history and physical examination. All enrolled patients received IV Akynzeo^®^ (fosnetupitant 235 mg and palonosetron 0.25 mg) diluted up to 50 mL with either 30 mL of 0.9% sodium chloride or 5% glucose and infused over 30 minutes (initiated 30 minutes before chemotherapy and completed before the start of chemotherapy).

The overall observation period was 10 days (±2 days). On Day 1, patients received CINV prophylaxis with dexamethasone at the oncologist’s discretion, followed by nine days of telephonic follow-up to evaluate safety and effectiveness. Assessments were conducted on Day 2 (24 hours), Day 5 (120 hours), and Day 10 (±2 days; 240 hours). Data collected included the timing and duration of each emetic episode, severity of nausea, use of rescue medications, and any adverse events (AEs).

The severity of nausea was assessed using a Visual Analogue Scale (VAS) ranging from 0 to 100 mm: VAS <5 mm, no nausea; VAS 5 to <25 mm, no significant nausea; VAS 25-74 mm, moderate nausea; and VAS 75-100 mm, severe nausea. Safety monitoring included recording treatment-emergent AEs (TEAEs) and serious TEAEs (STEAEs) during all telephonic and in-person follow-ups, along with completion of effectiveness and symptom questionnaires for AE evaluation.

Inclusion and exclusion criteria

Adult patients (male or female) aged ≥18 and ≤75 years, scheduled to receive their first cycle of chemotherapy, willing to provide written informed consent, and available for follow-up were included. Patients were excluded if they had a history of serious cardiovascular disease or predisposition to cardiac conduction abnormalities, were receiving medications that could cause conduction abnormalities (as per the treating physician’s assessment), had vomiting, retching, or more than insignificant nausea within 24 hours prior to informed consent, or required multiday chemotherapy.

Study endpoints

The primary endpoints were the number of patients with drug-related TEAEs (as assessed by the treating physician), the number of patients with TEAEs, and the number of patients with STEAEs, evaluated up to 10 days (240 hours).

Secondary endpoints included complete response (no emesis and no rescue medication), complete protection (no emesis, no rescue medication, and no significant nausea), and complete control (no emesis, no rescue medication, and no nausea). These were assessed during the acute phase (up to 24 hours), delayed phase (24-120 hours), extended phase (120-240 hours), overall phase (0-120 hours), and extended overall phase (0-240 hours, i.e., 10 days). Nausea severity was also assessed using the VAS up to 240 hours (10 days).

The study was conducted in accordance with the study protocol, the New Drugs and Clinical Trials Rules 2019 issued by the Government of India, the ethical principles of the Declaration of Helsinki (64th World Medical Association General Assembly, Fortaleza, Brazil, October 2013), the International Council for Harmonisation (ICH) Good Clinical Practice (GCP) guidelines, and all applicable local regulatory requirements.

Statistical analysis

Data were analyzed using appropriate statistical techniques. Continuous variables were summarized using descriptive statistics (mean, median, SD, minimum, and maximum), while categorical variables were summarized as the number and percentage of patients in each category. Effectiveness endpoints were primarily analyzed using descriptive methods. The 95% CI for the proportion of patients was calculated using binomial exact approximation.

## Results

**Figure 1:**
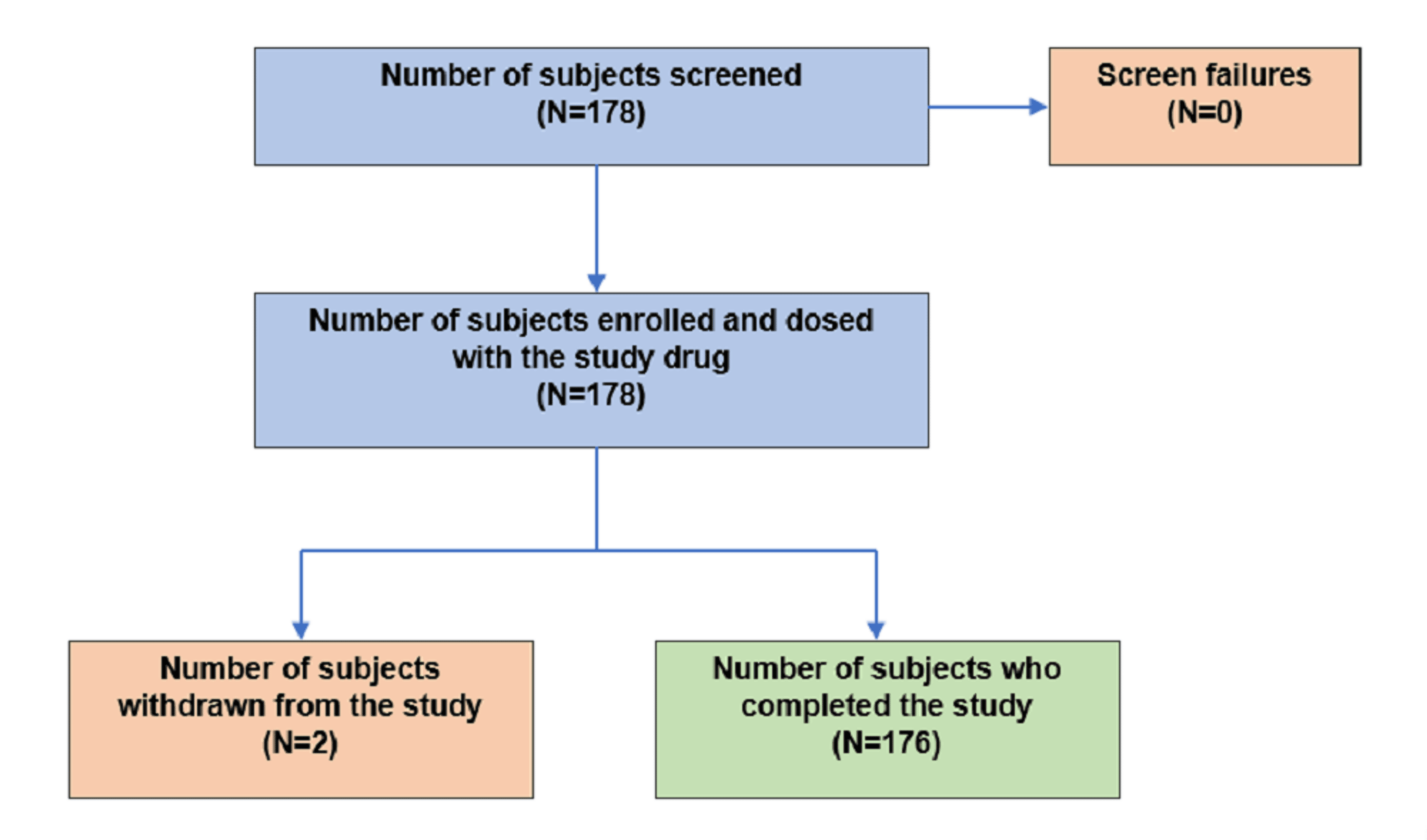
Study design

Of the 178 patients enrolled, 176 completed the study as per protocol, while two patients discontinued: one was lost to follow-up, and one died (as determined by the investigator, due to underlying disease progression and not related to the study drug) (Figure 1).

All 178 screened patients were enrolled. The majority were male (114; 64.04%), and the median age of the overall population was 48.5 years (Table 1).

**Table 1:** Baseline patient characteristics HEC, highly emetogenic chemotherapy; MEC, moderately emetogenic chemotherapy

Parameter	Variable	Number of patients, n (%)
Gender	Male	114 (64.04)
	Female	64 (35.96)
Prone to motion sickness	Yes	7 (3.93)
	No	171 (96.07)
History of morning sickness during pregnancy	Yes	1 (0.56)
	No	63 (35.39)
Alcohol use	Little/none	165 (92.70)
	Moderate/severe	13 (7.30)
Physician-perceived patient anxiety	Yes	7 (3.93)
	No	171 (96.07)
Type of chemotherapy received	MEC	88 (49.44)
	HEC	90 (50.56)

A total of 17 patients (9.55%) reported 23 AEs. None were considered related to IV Akynzeo^®^. Of these, 22 were mild in nature and did not require treatment, while 1 was reported as a serious/fatal AE (Table 2).

**Table 2:** Overall AEs AE, adverse event

Parameter	Variable	Number of AEs, n (%)
Severity (n = 23)	Mild	22 (95.65)
	Severe	1 (4.35)
Serious AE (n = 23)	Yes	1 (4.35)
	No	22 (95.65)
Relationship to study drug (n = 23)	Related	0 (0)
	Not related	23 (100)
Outcome (n = 23)	Recovered	22 (95.65)
	Not recovered (fatal)	1 (4.35)

The most commonly reported AEs were headache in four patients (2.25%); fatigue and fever in three patients each (1.69%); and infusion site reactions (pain and swelling) in three patients (1.68%). Other reported AEs, each occurring in one patient (0.56%), included bleeding, body aches, breathing difficulty, chest pain, chills, cough, loose motion, low appetite, and peripheral sensory neuropathy. All of these events were considered unrelated to the study drug.

**Figure 2:**
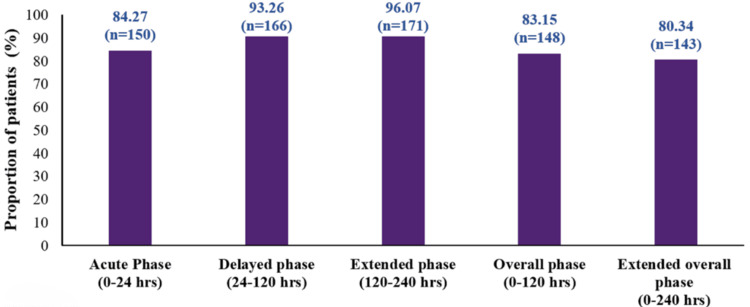
Complete response rate

The complete response rate was observed in 84.27% of patients (150/178; 95% CI: 78.07-89.29) during the acute phase; 93.26% (166/178; 95% CI: 88.52-96.47) during the delayed phase; 96.07% (171/178; 95% CI: 92.07-98.40) during the extended phase; 83.15% (148/178; 95% CI: 76.82-88.33) during the overall phase; and 80.34% (143/178; 95% CI: 73.73-85.91) during the extended overall phase (Figure 2).

**Figure 3:**
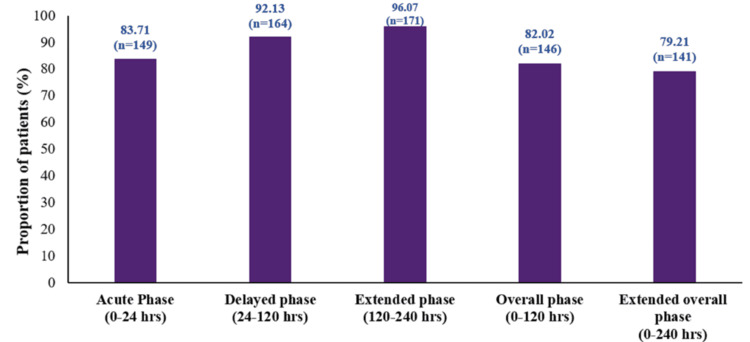
Complete protection rate

The complete protection rate was observed in 83.71% of patients (149/178; 95% CI: 77.45-88.81) during the acute phase; 92.13% (164/178; 95% CI: 87.16-95.63) during the delayed phase; 96.07% (171/178; 95% CI: 92.07-98.40) during the extended phase; 82.02% (146/178; 95% CI: 75.58-87.37) during the overall phase; and 79.21% (141/178; 95% CI: 72.51-84.92) during the extended overall phase (Figure 3).

**Figure 4:**
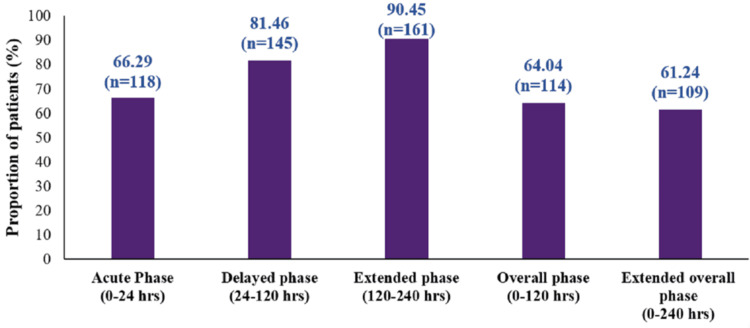
Complete control rate

The complete control rate was observed in 66.29% of patients (118/178; 95% CI: 58.84-73.19) during the acute phase; 81.46% (145/178; 95% CI: 74.96-86.88) during the delayed phase; 90.45% (161/178; 95% CI: 85.15-94.34) during the extended phase; 64.04% (114/178; 95% CI: 56.53-71.09) during the overall phase; and 61.24% (109/178; 95% CI: 53.66-68.43) during the extended overall phase (Figure 4).

## Discussion

Guidelines for the prevention of CINV recommend combination prophylaxis with agents targeting different pathways in patients scheduled to receive HEC or MEC. The American Society of Clinical Oncology, the Multinational Association of Supportive Care in Cancer/European Society of Medical Oncology, and the National Comprehensive Cancer Network recommend the use of NK1 RAs with 5-HT3 RAs and dexamethasone (triple combination) for patients receiving HEC regimens [14-17]. For patients receiving MEC, MASCC recently updated its guidance to recommend triple therapy prophylaxis in specific cases, including those receiving carboplatin (area under the curve ≥ 5), women < 50 years of age receiving oxaliplatin-based treatment, and patients treated with trastuzumab deruxtecan or sacituzumab govitecan. NEPA, which targets the two main emetic pathways, offers effective CINV prophylaxis and an opportunity to improve guideline-concordant antiemetic prescribing, thereby enhancing adherence with convenient administration [18]. IV Akynzeo^®^ (fosnetupitant 235 mg and palonosetron 0.25 mg), a concentrate for solution for infusion, is administered about 30 minutes before chemotherapy [19]. In addition to ensuring patient adherence, it provides an alternative route of administration for patients unable to tolerate oral therapy or with swallowing difficulties [1,14].

As a class, NK1 RAs are generally well tolerated [14]. Among them, injectable fosaprepitant, a commonly used agent, is associated with a higher incidence of injection site reactions, likely due to the surfactant polysorbate 80, particularly in patients receiving anthracycline-based chemotherapy. This can necessitate treatment interruption, switching to oral aprepitant, or changing injection sites, issues that represent an unmet clinical need [14]. In contrast, the low incidence of injection site reactions and hypersensitivity reported with IV NEPA is promising. This benefit is attributed to the distinctive chemical properties of fosnetupitant and the streamlined formulation of IV NEPA, which eliminates the need for surfactants, emulsifiers, solubility enhancers, or allergenic excipients [14,20,21]. Fosnetupitant has shown a minimal risk of infusion site reactions not only in patients receiving cisplatin but also in those treated with doxorubicin-cyclophosphamide/epirubicin-cyclophosphamide (AC/EC) regimens, which are particularly prone to such reactions [20]. In a Japanese study, the incidence of treatment-related injection site reactions was 0.3% with fosnetupitant compared with 3.6% with fosaprepitant (P < 0.001) [22]. Our study demonstrated a similarly low incidence of injection site reactions (1.68%) with IV NEPA, which were considered unrelated to the drug, supporting its potential to mitigate the risks associated with fosaprepitant administration.

The combination of fosnetupitant with palonosetron and dexamethasone presents a minimal risk of infusion site reactions while providing significant antiemetic efficacy, particularly during the extended delayed phase (120-168 hours), making it a convenient antiemetic therapy option [3]. In more than 1,300 cycles, IV NEPA demonstrated a favorable safety profile, with no infusion-site AEs or drug-related hypersensitivity reactions reported during the 120 hours following infusion [14,23]. Key studies on NEPA confirm its efficacy and position it as a promising choice for the efficient control of nausea [1]. In a head-to-head comparison, Hata et al. demonstrated that fosnetupitant was effective in controlling acute, delayed, and extended delayed CINV, with a favorable safety profile and reduced risk of injection site reactions [22].

In a phase IIIb, multinational, randomized, double-blind study, Schwartzberg et al. evaluated IV NEPA in female patients with breast cancer receiving anthracycline-cyclophosphamide (AC)-based chemotherapy who were new to HEC or MEC regimens [23]. The study demonstrated both safety and high efficacy, with a complete response rate (no emesis/no rescue) of 73.0% during cycle 1 (0-120 hours), which was maintained across subsequent cycles [11].

The safety of a single dose of IV NEPA (fosnetupitant 235 mg plus palonosetron 0.25 mg) administered over 30 minutes before initial and subsequent cycles of HEC was further confirmed in a multinational, randomized, double-blind, sex- and country-stratified phase III study by Schwartzberg et al. This trial found that IV NEPA was well tolerated, with a safety profile comparable to that of oral NEPA in chemotherapy-naïve patients with solid tumors [23].

Indian studies reporting clinical experience with NK1 RAs are summarized in Table 3 [24-27].

**Table 3:** Experience with NK1 RAs in Indian studies HEC, highly emetogenic chemotherapy; MEC, moderately emetogenic chemotherapy; NEPA, netupitant + palonosetron; RA, receptor antagonist

Study	Antiemetic	Chemotherapy regimen	Complete response, %		
			Acute phase	Delayed phase	Overall
Vaswani et al. (2022) [24]	NEPA + dexamethasone (chemo-naïve patients in an Indian setting)	HEC and MEC	93	85.7	85.4
Vaswani et al. (2020) [25]	NEPA + dexamethasone (real-life study)	HEC and MEC	97.3/98.9	93.6/94.0	93.6/94.0
Kaushal et al. (2015) [26]	Palonosetron + dexamethasone + aprepitant vs. ondansetron + dexamethasone (head and neck cancer)	Docetaxel, carboplatin, and 5-FU	86.7 vs. 60.0	83.3 vs. 53.3	83.3 vs. 53.3
Maru et al. (2013) [27]	IV fosaprepitant (Day 1) vs. oral aprepitant (three-day regimen) with ondansetron + dexamethasone (solid malignancies)	Cisplatin	94.2 vs. 90.1	77.7 vs. 73.9	77.1 vs. 73.4

In this study, IV Akynzeo^®^ was well tolerated, and nausea was the least severe in the majority of patients across all phases for both HEC and MEC. Antiemetic potency, measured by complete response, complete protection, and complete control rates, was particularly notable in the extended phase (120-240 hours), demonstrating substantial efficacy during the most difficult-to-control period of highest emetogenic risk. Additionally, the high complete response rates observed during the acute, delayed, and overall phases (ranging from 80.34% to 93.26%) provide further evidence supporting effective prevention of both acute and delayed CINV following the use of highly or moderately emetogenic chemotherapeutic agents.

Although occasional short bursts of nausea occurred, the results indicated that vomiting could be successfully prevented for 10 days without additional rescue medication when IV Akynzeo^®^ was administered prior to MEC or HEC cycles.

Treatment tolerance was good. A single fatal serious AE occurred during follow-up; the investigator judged this event to be unrelated to the study medication and attributed it to disease progression from carcinoma of the supraglottic larynx. The majority of AEs were consistent with the known effects of NK1 and 5-HT3 antagonist drug classes and chemotherapy side effects; they were generally mild and self-limited. No evidence of increased toxicity, higher event rates, severe toxicity, or unexpected safety concerns was observed compared with the established safety profiles of these agents. The study did not identify any new potential safety signals, supporting an acceptable safety profile for IV Akynzeo^®^.

IV Akynzeo^®^ (fosnetupitant 235 mg plus palonosetron 0.25 mg) demonstrated safety and effectiveness in this real-world study, consistent with findings from pivotal clinical trials. However, several limitations warrant acknowledgment. The single-arm, open-label design without a control group limited the ability to perform comparative assessments versus other antiemetic regimens or standard care. The lack of blinding may have introduced bias in subjective endpoints, particularly nausea severity measured by the VAS. Conducting the study exclusively at six Indian sites may limit generalizability to other ethnic groups or healthcare settings with different practices and patient demographics. The sample size (n = 178), while appropriate for a phase IV study, may have been insufficient to detect rare AEs or subtle efficacy differences among subgroups. Reliance on telephonic follow-up for most assessments (Days 2, 5, and 10) could have affected data completeness and quality compared with in-person evaluation, especially for subjective measures. The study population was limited to patients receiving first-cycle HEC/MEC chemotherapy, which may not reflect real-world situations in which patients receive multiple cycles. Finally, as a post-marketing phase IV study, the design lacked the stricter controls of earlier randomized trials, which may affect the robustness of conclusions about comparative effectiveness and safety.

## Conclusions

IV Akynzeo^®^ was highly effective in preventing acute, delayed, and extended delayed CINV in cancer patients receiving HEC and MEC regimens. The combination demonstrated compelling effectiveness and a favorable safety profile, supporting its use as a preferred option for the prevention of CINV. IV Akynzeo^®^ provides a simple and convenient CINV prophylaxis option, addressing symptoms for up to 10 days after chemotherapy with a single dose administered on Day 1.

## References

[1] Aapro M, Zhang L, Yennu S, LeBlanc TW, Schwartzberg L (2019). Preventing chemotherapy-induced nausea and vomiting with netupitant/palonosetron, the first fixed combination antiemetic: current and future perspective. Future Oncol.

[2] Roeland E, Aapro MS, Schwartzberg LS (2015). Advances in the management of chemotherapy-induced nausea and vomiting: new data from recent and ongoing trials. Clin Adv Hematol Oncol.

[3] Abe M, Iihara H, Aogi K (2023). Fosnetupitant for the prevention of chemotherapy-induced nausea and vomiting: a short review and clinical perspective. Adv Ther.

[4] Vaid AK, Gupta S, Doval DC (2020). Expert consensus on effective management of chemotherapy-induced nausea and vomiting: an Indian perspective. Front Oncol.

[5] Aapro M (2018). CINV: still troubling patients after all these years. Support Care Cancer.

[6] Adel N (2017). Overview of chemotherapy-induced nausea and vomiting and evidence-based therapies. Am J Manag Care.

[7] Rapoport BL (2017). Delayed chemotherapy-induced nausea and vomiting: pathogenesis, incidence, and current management. Front Pharmacol.

[8] Ballatori E, Roila F, Ruggeri B, Bruno AA, Tiberti S, di Orio F (2010). The burden of chemotherapy induced nausea and vomiting on patients’ daily lives: Italian perspectives. Handbook of Disease Burdens and Quality of Life Measures.

[9] Sun Y, Zheng Y, Yang X (2021). Incidence of chemotherapy-induced nausea and vomiting among cancer patients receiving moderately to highly emetogenic chemotherapy in cancer centers in Sichuan, China. J Cancer Res Clin Oncol.

[10] Chow R, Yin LB, Baqri W (2023). Prevalence and predictors of long-delayed (> 120 h) chemotherapy-induced nausea and vomiting (CINV)—a systematic review and individual patient data meta-analysis. Support Care Cancer.

[11] Schwartzberg L, Navari R, Clark-Snow R (2020). Phase IIIb safety and efficacy of intravenous NEPA for prevention of chemotherapy-induced nausea and vomiting (CINV) in patients with breast cancer receiving initial and repeat cycles of anthracycline and cyclophosphamide (AC) chemotherapy. Oncologist.

[12] List of new drugs approved in year 2018. https://cdsco.gov.in/opencms/resources/UploadCDSCOWeb/2018/UploadApprovalNewDrugs/latesnd18approval.pdf.

[13] Aapro M, Caprariu Z, Chilingirov P (2022). Assessing the impact of antiemetic guideline compliance on prevention of chemotherapy-induced nausea and vomiting: Results of the nausea/emesis registry in oncology (NERO). Eur J Cancer.

[14] Aapro M, Navari RM, Roeland E, Zhang L, Schwartzberg L (2021). Efficacy of intravenous NEPA, a fixed NK1/5-HT3 receptor antagonist combination, for the prevention of chemotherapy-induced nausea and vomiting (CINV) during cisplatin- and anthracycline cyclophosphamide (AC)-based chemotherapy: a review of phase 3 studies. Crit Rev Oncol Hematol.

[15] (2021). Errata. J Clin Oncol.

[16] Roila F, Warr D, Hesketh PJ (2017). Erratum to: 2016 updated MASCC/ESMO consensus recommendations: prevention of nausea and vomiting following moderately emetogenic chemotherapy. Support Care Cancer.

[17] NCCN Clinical Practice Guidelines in Oncology (NCCN Guidelines®): antiemesis (version 1.2021). https://www.nccn.org.

[18] Aapro M, Jordan K, Scotté F, Celio L, Karthaus M, Roeland E (2022). Netupitant-palonosetron (NEPA) for preventing chemotherapy-induced nausea and vomiting: from clinical trials to daily practice. Curr Cancer Drug Targets.

[19] (2025). Akynzeo® US Prescribing Information. https://www.akynzeo.com/assets/pdf/Akynzeo-USPI.pdf.

[20] Matsuura K, Tsurutani J, Inoue K (2022). A phase 3 safety study of fosnetupitant as an antiemetic in patients receiving anthracycline and cyclophosphamide: CONSOLE-BC. Cancer.

[21] Fujii T, Nishimura N, Urayama KY (2015). Differential impact of fosaprepitant on infusion site adverse events between cisplatin- and anthracycline-based chemotherapy regimens. Anticancer Res.

[22] Hata A, Okamoto I, Inui N (2022). Randomized, double-blind, phase III study of fosnetupitant versus fosaprepitant for prevention of highly emetogenic chemotherapy-induced nausea and vomiting: CONSOLE. J Clin Oncol.

[23] Schwartzberg L, Roeland E, Andric Z (2018). Phase III safety study of intravenous NEPA: a novel fixed antiemetic combination of fosnetupitant and palonosetron in patients receiving highly emetogenic chemotherapy. Ann Oncol.

[24] Vaswani B, Dattatreya PS, Barkate H, Bhagat SB, Patil S, Jadhav AY (2022). The effectiveness of an oral fixed-dose combination of netupitant and palonosetron (NEPA) in patients with multiple risk factors for chemotherapy-induced nausea and vomiting: a multicenter, observational indian study. Cureus.

[25] Vaswani B, Bhagat S, Patil S, Barkate H (2020). Effectiveness of a novel, fixed dose combination of netupitant and palonosetron in prevention of chemotherapy induced nausea and vomiting: a real-life study from India. World J Clin Oncol.

[26] Kaushal P, Atri R, Soni A, Kaushal V (2015). Comparative evaluation of triplet antiemetic schedule versus doublet antiemetic schedule in chemotherapy-induced emesis in head and neck cancer patients. Ecancermedicalscience.

[27] Maru A, Gangadharan VP, Desai CJ, Mohapatra RK, Carides AD (2013). A phase 3, randomized, double-blind study of single-dose fosaprepitant for prevention of cisplatin-induced nausea and vomiting: results of an Indian population subanalysis. Indian J Cancer.

